# P-1196. Drug-Drug Interactions (DDIs) With Letermovir (LET) for Cytomegalovirus (CMV) Prophylaxis in Pediatric (Birth to < 18 years of age) Hematopoietic Cell Transplant (HCT) Recipients

**DOI:** 10.1093/ofid/ofae631.1380

**Published:** 2025-01-29

**Authors:** Lara A Danziger-Isakov, Andreas H Groll, Natasha Broyde, Barbara A Haber, Christopher L Gilbert, Cyrus Badshah

**Affiliations:** Cincinnati Children's Hospital, Cincinnati, Ohio; University Children’s Hospital Muenster, Muenster, Germany, Muenster, Nordrhein-Westfalen, Germany; Merck & Co., Inc., Kenilworth, NJ; Merck & Co., Inc., Kenilworth, NJ; Merck & Co., Inc., Kenilworth, NJ; Merck & Co., Inc., Kenilworth, NJ

## Abstract

**Background:**

HCT recipients receive multiple medications post-transplant, increasing the potential for DDIs. LET, a CMV terminase complex inhibitor approved for CMV prophylaxis in adult CMV-seropositive allogeneic HCT recipients, has the potential for DDIs with multiple medications commonly used in the HCT setting. As LET-associated DDIs have been well characterized in adults, the objective of this analysis was to evaluate known LET-associated DDIs in pediatric HCT recipients, including impact on patient safety, in a recently completed Phase 2 nonrandomized, multicenter, open-label study (NCT03940586) which evaluated LET prophylaxis in pediatric allo-HCT recipients.

**Methods:**

Participants from birth to < 18 years of age were enrolled within 28 days post-HCT to receive LET through 14 weeks (∼100 days) post-HCT and followed through Week 48 post-HCT. Medications with potentially significant drug interactions (per the LET US label) administered concomitantly with LET were assessed in all study participants.

**Results:**

A total of 63 enrolled participants received at least one dose of LET. Sixty-two (98.4%) participants received one or more concomitant medications with a potentially significant LET-associated DDI, and 50 (79.4%) participants received two or more LET-associated DDI medications. The most common concomitant medications with a LET-associated DDI were cyclosporin A (60.3% [38/63]), tacrolimus (41.3%), and voriconazole (39.7%). DDIs were reported as an adverse event (AE) in five (7.9%) participants: cyclosporin A increased (n=2), voriconazole decreased (n=2), and tacrolimus interaction (n=1). LET was discontinued in one participant due to decreased voriconazole levels. No AEs or deaths were reported due to these DDI events.

**Conclusion:**

Concomitant administration of LET with medications with known potentially significant LET-associated DDIs was generally safe and well tolerated in pediatric HCT recipients.

**Disclosures:**

**Lara A. Danziger-Isakov, MD, MPH**, Aicuris: clinical research contract, paid to institutio|Ansun BioPharma: clinical research contract, paid to institution|Astellas: Advisor/Consultant|Astellas: clinical research contract, paid to institutio|Merck: clinical research contract, paid to institutio|Pfizer: Grant/Research Support|Takeda: clinical research contract, paid to institutio **Andreas H. Groll, MD**, Amplyx: Advisor/Consultant|Astellas: Advisor/Consultant|Astellas: Speaker bureau|Basilea: Advisor/Consultant|Basilea: Speaker bureau|F2G: Advisor/Consultant|F2G: Speaker bureau|Gilead Sciences: Advisor/Consultant|Gilead Sciences: Grant/Research Support|Gilead Sciences: Speaker bureau|Merck Sharp & Dohme LLC: Advisor/Consultant|Merck Sharp & Dohme LLC: Grant/Research Support|Merck Sharp & Dohme LLC: Speaker bureau|Mundipharma: Grant/Research Support|Pfizer: Advisor/Consultant|Pfizer: Grant/Research Support|Pfizer: Speaker bureau|Subsidiary of Merck & Co: Advisor/Consultant|Subsidiary of Merck & Co: Grant/Research Support|Subsidiary of Merck & Co: Speaker bureau **Natasha Broyde, MS**, Merck Sharp & Dohme LLC,: Current employee|Merck Sharp & Dohme LLC,: Stocks/Bonds (Public Company) **Barbara A. Haber, MD**, Merck & Co., Inc: Employee of Merck & Co., Inc **Christopher L. Gilbert, n/a**, Merck Sharp & Dohme LLC: Current employee|Merck Sharp & Dohme LLC: Stocks/Bonds (Public Company) **Cyrus Badshah, MD, PhD**, Merck Sharp & Dohme LLC: Current employee|Merck Sharp & Dohme LLC: Stocks/Bonds (Public Company)

